# Short-term carcinogenicity study of N-methyl-N-nitrosourea in FVB-Trp53 heterozygous mice

**DOI:** 10.1371/journal.pone.0280214

**Published:** 2023-01-06

**Authors:** Na-Won Kim, Sun-Min Seo, Eun-Seon Yoo, Ah-Reum Kang, Ji-Hun Lee, Jae-Hoon Lee, Byeong-Cheol Kang, Han-Woong Lee, Yang-Kyu Choi

**Affiliations:** 1 Department of Laboratory Animal Medicine, College of Veterinary Medicine, Konkuk University, Seoul, Republic of Korea; 2 Department of Biochemistry, College of Life Science and Biotechnology, Yonsei University, Seoul, Republic of Korea; 3 Graduate School of Translational Medicine, Seoul National University College of Medicine, Seoul, Republic of Korea; Bose Institute, INDIA

## Abstract

Carcinogenicity tests predict the tumorigenic potential of various substances in the human body by studying tumor induction in experimental animals. There is a need for studies that explore the use of FVB/N-Trp53^em2Hwl^/Korl (FVB-Trp53^+/-^) mice, created by TALEN-mediated gene targeting in Korea, in carcinogenicity tests. This study was performed to determine whether FVB-Trp53^+/-^ mice are a suitable model for short-term carcinogenicity studies. To compare the carcinogenicity at different concentrations, 25, 50, and 75 mg/kg of N-methyl-N-nitrosourea (MNU), a known carcinogen, were administered intraperitoneally to FVB-Trp53^+/-^ and wild-type male mice. After 26 weeks, the survival rate was significantly reduced in FVB-Trp53^+/-^ mice compared to the wild-type mice in the 50 and 75 mg/kg groups. The incidence of thymic malignant lymphoma (TML) in the 50 and 75 mg/kg groups was 54.2 and 59.1% in FVB-Trp53^+/-^ male mice, respectively. TML metastasized to the lungs, spleen, lymph nodes, liver, kidney, and heart in FVB-Trp53^+/-^ male mice. Furthermore, the incidence of primary lung tumors, such as adenomas and adenocarcinomas, was 65.4, 62.5, and 45.4% in the FVB-Trp53^+/-^ mice of the 25, 50, and 75 mg/kg groups, respectively. The main tumor types in FVB-Trp53^+/-^ mice were TML and primary lung tumors, regardless of the dose of MNU administered. These results suggest that systemic tumors may result from malfunctions in the p53 gene and pathway, which is an important factor in the pathogenesis of human cancers. Therefore, FVB-Trp53 heterozygous mice are suitable for short-term carcinogenicity tests using positive carcinogens, and that the best result using MNU, a positive carcinogen, might have a single dose of 50 mg/kg.

## Introduction

Carcinogenicity tests use experimental animals to predict the risk of tumorigenesis from exposure to various substances [1]. These tests can be performed for drugs suspected of having carcinogenic effects or for drugs that repeatedly caused lesions in dose toxicity tests [2]. Carcinogenicity tests are classified into long- and short-term tests. Compared to long-term carcinogenicity tests, short-term carcinogenicity tests use fewer laboratory animals, last a shorter period of time, and cost lesser [3].

p53 is a tumor suppressor gene related to oncogenic signaling pathways [4, 5]. p53 heterozygous mice mainly develop soft-tissue sarcoma, osteosarcoma, and lymphoma, which show a very similar carcinogenesis pattern to patients with Li-Fraumeni syndrome, caused by p53 germline mutation [6]. p53 heterozygous mice have been used as models for lung, brain, and bone tumors, lymphoma, and leukemia [7].

Mutations in p53 gene have been observed in 50% of cancer patients [8, 9]. The p53 gene can stop the cell cycle and division and regulate DNA recovery and immune response [10]. The deactivation of this gene is frequently observed in patients with lung cancer. Upregulation or activation of p53 can inhibit the progression of lung tumors [11]. p21, which is downstream to p53, is responsible for inhibiting the G1 phase, while other p53 target genes are responsible for inhibiting the G2 cell cycle [12].

The most commonly used positive carcinogens in the p53 heterozygous model are p-cresidine [13] and N-methyl-N-nitrosourea (MNU) [14]. MNU can induce the development of various tumors in multiple organs depending on the animal species, strain, age, dosage, and route of administration [15], including many types of mammary tumors and thymic lymphomas [16].

C57BL/6 background Trp53^+/-^ mice are widely used in carcinogenicity tests. When 75 mg/kg MNU is administered intraperitoneally to B6-Trp53 heterozygous mice, malignant lymphoma occur in two main target organs; 100% in the thymus and spleen [17]. The main organs where lymphomas metastasize are the thymus, spleen, bone marrow, and lymph nodes [17, 18]. The incidence of lung adenoma in B6-Trp53 heterozygous mice was significantly higher than that in wild-type mice. Tumors appearing in the thymus were diagnosed as malignant lymphoma, which occurred with a higher probability in p53 heterozygous mice than wild-type mice. In addition, rhabdomyosarcoma, leiomyosarcoma, malignant schwannoma, and sarcoma have been reported in p53 heterozygous mice [19]. Recently, the FVB/N-Trp53^em2Hwl^/Korl (FVB-Trp53) mouse was created using TALEN-mediated gene targeting in Korea [20]. This study was conducted to determine whether the newly developed mouse model in Korea is suitable for a short-term carcinogen test using MNU, a positive carcinogen, in FVB-Trp53 mice.

## Materials and methods

### Chemical and dose formulation

N-methyl-N-nitrosourea (MNU, CAS No. 684-93-5, Spectrum, USA) was dissolved in citrate-buffered saline prepared at pH 4.5 immediately before use. MNU was prepared at doses of 25, 50, and 75 mg/kg per body weight and administered via intraperitoneal (IP) injection.

### Animals and treatments

In this study, 6-week-old male FVB-Trp53^+/-^ and wild-type mice were used. Both mice were obtained from Yonsei University, Seoul, Republic of Korea [20]. FVB-Trp53^+/-^ and wild-type mice were housed in a laboratory animal facility at the College of Veterinary Medicine, Konkuk University. After acclimatization in the animal room for 7 days, FVB-Trp53^+/-^ and wild-type mice were randomly divided into four groups according to body weight: 0, 25, 50, and 75 mg/kg. The facility was maintained under conditions free of specific pathogens using a barrier system. The strains were bred in individually ventilated cages with sterile feed, water, and bedding. The facility was maintained in an air-conditioned system at 22 ± 2°C, at a relative humidity of 50 ± 10%, and a 12 h light /12 h dark cycle. To diminish distress, pulp house and wood chew block were provided for mice. The mice were weighed once a week to check for weight changes by trained researchers. The activity, appearance, and survival of the mice were observed once daily. Humane euthanasia performed on the day reaching 25% of activity score. Mice found dead were necropsied immediately. All animal experiments were approved by the Institutional Animal Care and Use Committee of Konkuk University, Korea (KU20081).

### Hematology and serum chemistry

At 26 weeks, the mice were fasted overnight and anesthetized with isoflurane, and blood samples were collected from the caudal vena cava. General hematological tests included white blood cell (WBC) count, red blood cell (RBC) count, hemoglobin, hematocrit, mean corpuscular volume (MCV), mean corpuscular hemoglobin (MCH), mean corpuscular hemoglobin concentration (MCHC), cellular hemoglobin concentration mean (CHCM), red cell distribution width (RDW), hemoglobin distribution width (HDW), cellular hemoglobin content (CH), and cell hemoglobin distribution width (CHDW). Platelets, mean platelet volume (MPV), platelet distribution width (PDW), platelet count, differential leukocyte count (absolute and relative), large unstained cells (LUC, absolute and relative), and reticulocytes (absolute and relative) were tested using an Animal Blood counter (ADVIA 2120i, Siemens Healthcare Diagnostics Ltd., Ireland).

For serum collection, the collected blood was left at room temperature for 30 min to coagulate, and then centrifuged for 15 min at 3,000 rpm. Alanine aminotransferase (ALT), alkaline phosphatase (ALP), aspartate aminotransferase (AST), γ-glutamyl transferase (γGT), triglyceride, albumin, glucose, albumin/globulin (A/G) ratio, total protein, total cholesterol (TC), total bilirubin, blood urea nitrogen (BUN), creatinine, BUN/creatinine (B/C) ratio, calcium, chlorine, inorganic phosphorus (IP), potassium, and sodium levels were measured using an automatic chemistry analyzer (Hitachi7070, HITACHI, Japan).

### Histopathological analysis

Except for unscheduled death, mice were sacrificed 26 weeks after MNU administration. The lungs, spleen, and liver were separated and weighed, and relative organ weights (organ weight to body weight ratio) were calculated. All organs collected for histopathological evaluation were fixed by embedding in 10% neutral-buffered formalin and processed to prepare paraffin blocks. The prepared paraffin blocks were cut into 4 μm sections and attached to slides. After deparaffinization, the slides were stained with hematoxylin and eosin. The prepared slides were observed under a BX51 microscope (Olympus, Japan) and analyzed using the DP71 (Olympus) program.

### Immunohistochemistry

Paraffin blocks were sliced to a thickness of 4 μm and attached to a silane-coated slide (Muto Pure Chemicals Co., Ltd., Japan). Each procedure was performed according to the ABC kit protocol (Vector Laboratories, USA). After deparaffinization and rehydration, slides were boiled in 0.1 M sodium citrate buffer (pH 6.0) in a microwave oven for antigen retrieval. After cooling the slides to room temperature, 3% H_2_O_2_ in methanol was used to block the endogenous peroxidase activity. To suppress non-specific reactions, blocking serum (Vector Laboratories) was applied to the tissue. PCNA antibody (Abcam, ab92552, diluted 1:200), prosurfactant protein C (SPC, Abcam, ab90716, diluted 1:1000), and ubiquitin antibody (CC10, Abcam, ab213203, diluted 1:4000) were used as the primary antibodies. Biotinylated antibody (Vector Laboratories, USA) was used as a secondary antibody. It was then detected using the DAB Peroxidase Substrate Kit (Vector Laboratories, USA). All the slides were counterstained with hematoxylin (Gill III hematoxylin, Thermo, USA).

### Statistical analysis

For statistical analysis, GraphPad Prism 7.04 (GraphPad Software, USA) was used to perform two tailed *t*-test, long-rank test, and chi-square test. *P < 0.05, **P < 0.01, ***P < 0.001 were considered statistically significant.

## Results

### Body weight changes

**Fig 1:**
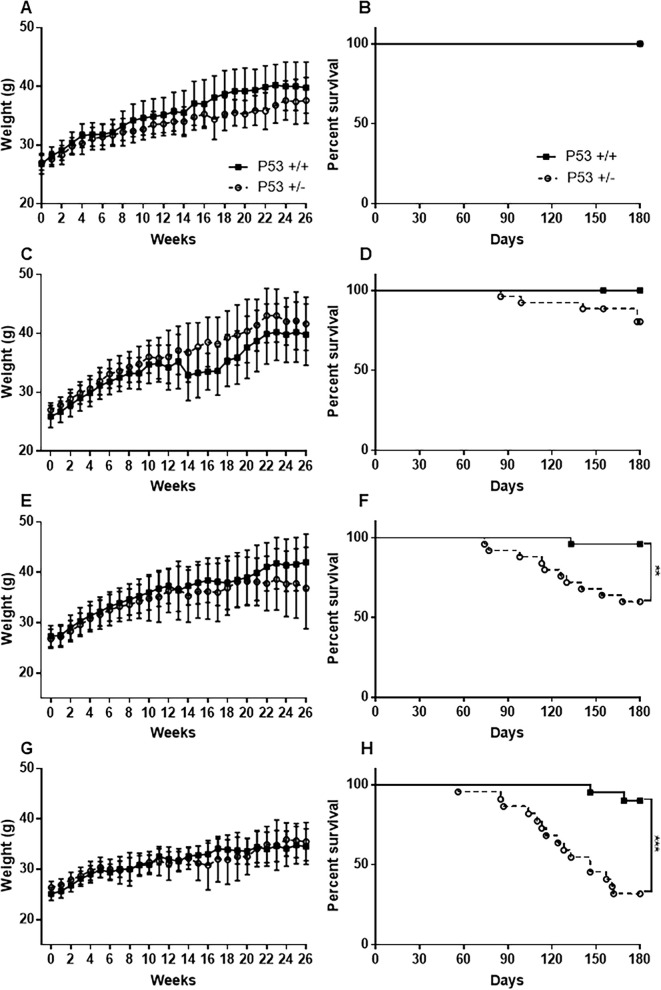
Body weight change and survival rate after MNU administration in different dosages. Body weight change of the 0 (untreated control; A), 25mg/kg (C), 50mg/kg (E), and 75mg/kg (G) group; survival rate of the 0 (B), 25mg/kg (D), 50mg/kg (F), and 75mg/kg (H) group. Data are represented as mean ± SD. **P < 0.01 and ***P < 0.001 versus wild-type mice, as assessed using *Long-rank* test.

The weights of mice in each group were measured once per week. The body weight of FVB-p53^+/-^ and wild-type mice gradually increased over time in the 0 (untreated control), 25, 50, and 75 mg/kg groups. There was no significant difference in body weight between heterozygous and wild-type mice in the same MNU administration group (Fig 1A, 1C, 1E and 1G).

### Mouse survival rate

At the end of the study at 26 weeks, the survival rate in the 25 mg/kg group was 84.6% (22 of 26 mice) in FVB-Trp53^+/-^ mice and 100% (19 of 19 mice) in wild-type mice (Fig 1D). The survival rate in the 50 mg/kg group was 62.5% (15 of 24 mice) in FVB-Trp53^+/-^ mice and 96.2% (25 of 26 mice) in wild-type mice (Fig 1F). The survival rate in the 75 mg/kg group was 31.8% (7 of 22 mice) in FVB-Trp53^+/-^ mice and 90% (18 of 20 mice) in wild-type mice (Fig 1H). The survival rates of 50 and 75 mg/kg groups in FVB-p53^+/-^ mice was significantly lower than those of wild-type mice (Fig 1F and 1H).

### Hematology and serum chemistry

Thirty hematology tests and 19 serum chemistry tests were performed. Hematological analysis revealed that the percentage of neutrophils in the 25 mg/kg group was significantly higher in FVB-Trp53^+/-^ than in wild-type mice. In the 50 mg/kg group, the number of RBCs and neutrophils and the percentage of neutrophils were significantly higher in FVB-Trp53^+/-^ than in wild-type mice; however, MCH and the percentage of lymphocytes were significantly lower in FVB-Trp53^+/-^ than in wild-type mice. In the 75 mg/kg group, MCV, MPV, and PDW were significantly lower in FVB-Trp53^+/-^ than in wild-type mice (S1 Table). Some hematology tests exhibited a significant difference between the groups; however, all results were within the reference range.

The results of the serum chemistry tests in the 25 mg/kg group show that IP significantly increased and triglyceride significantly decreased in FVB-Trp53^+/-^ mice compared to wild-type mice. In the 75 mg/kg group, glucose and IP were significantly higher in FVB-Trp53^+/-^ mice than in wild-type mice; however, calcium levels were significantly lower in FVB-Trp53^+/-^ mice than in wild-type mice (S2 Table). Similar to the hematological analysis, some serum chemistry tests exhibited a significant difference between groups; however, all results were within the reference range.

### Organ to body weight ratio

**Fig 2:**
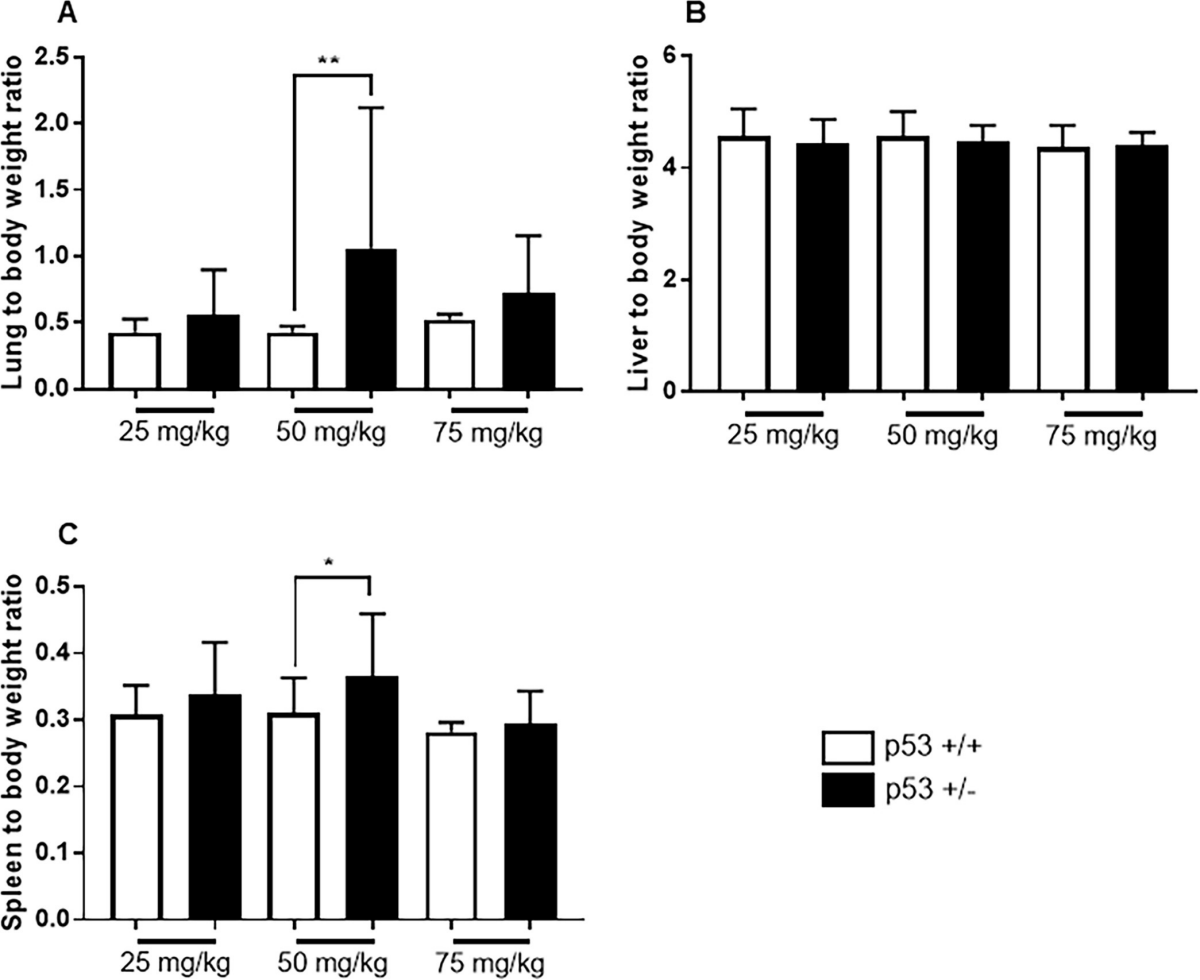
Relative organ weights after MNU administration in different dosages. (A) shows lung to body weight ratio, (B) shows liver to body weight ratio, and (C) shows spleen to body weight ratio in the mice survived until the end of the experiment. Data are represented as mean ± SD. *P < 0.05 and **P < 0.01 versus wild type mice, as assessed using two-tailed *t*-test.

Organ weights were measured in all surviving mice until 26 weeks after MNU administration. In the 25 and 75 mg/kg groups, no significant difference was observed between the lung, liver, and spleen weights (%) of FVB-Trp53^+/-^ and wild-type mice. In the 50 mg/kg group, the relative lung and spleen weights of FVB-Trp53^+/-^ mice were significantly higher than those of wild-type mice (Fig 2).

### Gross lesion

**Fig 3:**
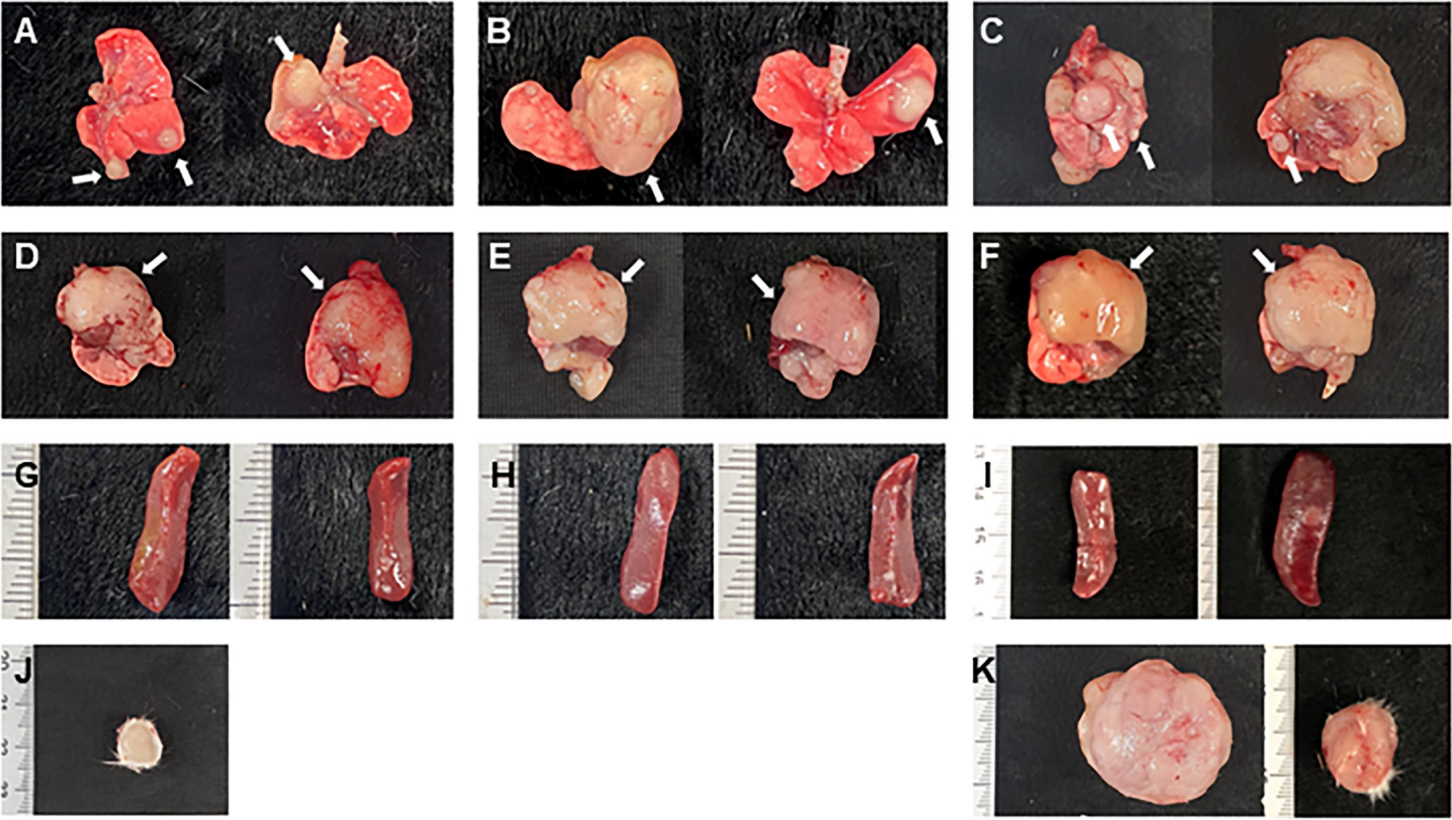
Representative images of gross lesions after MNU administration in different dosages. Macroscopic appearance of large mass (white arrows) in the lungs (A, B, C), and thymus (D, E, F) shown in FVB-p53^+/-^ mice of the 25mg/kg (A, D), 50mg/kg (B, E), and 75mg/kg (C, F) group. Splenomegaly (G, H, I) and a large subcutaneous mass (J, K) shown in the FVB-p53^+/-^ mice of the 25mg/kg (G, J), 50mg/kg (H), and 75mg/kg (I, K) group.

During the experiment, all moribund mice were sacrificed, and all surviving mice were necropsied 26 weeks after MNU administration. In FVB-Trp53^+/-^ mice, lung nodules were found in 76.9% of the 25 mg/kg group, 91.7% of the 50 mg/kg group, and 90.9% of the 75 mg/kg group (Fig 3A–3C). However, in wild type mice, lung nodules were found in 52.6%, 76.9% and 90% of the 25, 50, and 75 mg/kg groups, respectively.

In the 25 mg/kg group, enlarged thymus (Fig 3D) and splenomegaly (Fig 3G) were found in 11.5% and 7.7% of FVB-Trp53^+/-^ mice, respectively, while none were found in wild-type mice. In the 50 mg/kg group, enlarged thymus (Fig 3E) and splenomegaly (Fig 3H) were found in 54.2% and 29.2% of FVB-Trp53^+/-^ mice, respectively, while none were found in wild-type mice. In the 75 mg/kg group, enlarged thymus (Fig 3F) and splenomegaly (Fig 3I) were found in 59.1% and 27.3% of FVB-Trp53^+/-^ mice, respectively, and in only one wild-type mouse. Subcutaneous masses were observed in one FVB-Trp53^+/-^ mouse of the 25 mg/kg group (Fig 3J) and in three FVB-Trp53^+/-^ of the 75 mg/kg group (Fig 3K).

### Histopathology

**Fig 4:**
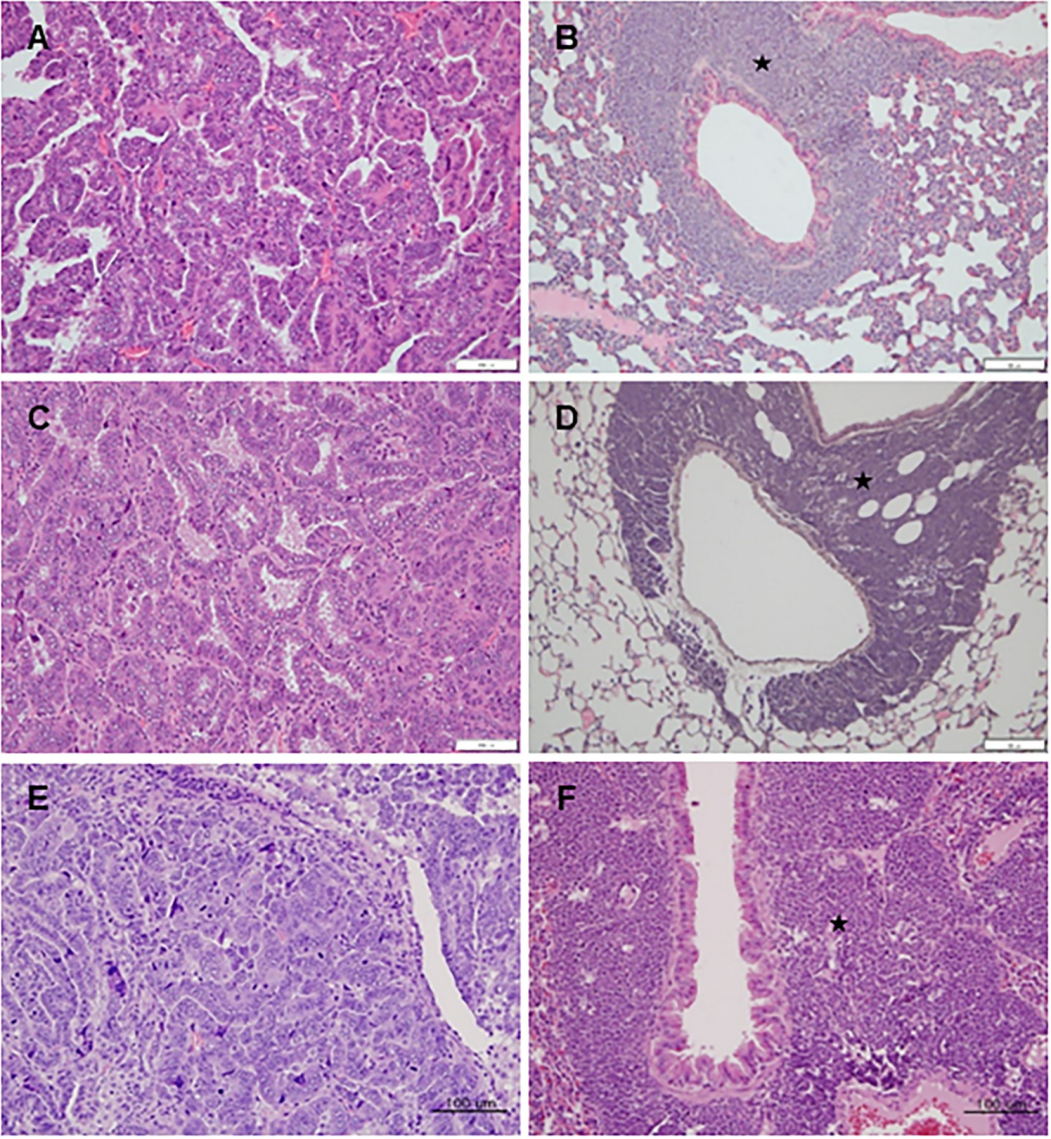
Representative images of histopathological lesions of adenocarcinoma and thymic malignant lymphoma (TML) in the lungs. (A), (C), and (E) show adenocarcinoma in the lungs of the FVB-p53^+/-^ mice of the 25, 50, and 75mg/kg group, respectively. (B), (D), and (F) show TML metastasis (black star) in the peribronchiolar or perivascular region of the FVB-p53^+/-^ mice of the 25, 50, and 75mg/kg group, respectively. Scale bar represents 100 *μ*m.

Based on tumor cell morphology observed during histopathological analysis of the lung nodules, primary lung tumors were divided into adenoma and adenocarcinoma (Fig 4A, 4C and 4E). In addition, histopathological analysis of enlarged thymus tissues revealed thymic malignant lymphoma (TML) as the primary tumor (Fig 4B, 4D and 4F). Metastatic TMLs were found in multiple organs, such as the lungs, liver, enlarged spleens, kidneys, lymph nodes, and heart (Table 1). TML metastasis in the lungs was mainly observed in the peribronchiolar or perivascular regions. Interestingly, two types of malignant tumors, primary lung adenocarcinoma and TML lung metastasis, were observed in the lungs of several mice, respectively (Table 1). The subcutaneous masses were identified as malignant fibrosarcomas.

**Table 1:** The number of tumors observed in each organ after MNU administration in different dosages.

	25 mg/kg		50 mg/kg		75 mg/kg	
	p53^+/+^	p53^+/-^	p53^+/+^	p53^+/-^	p53^+/+^	p53^+/-^
Total number of mice	19	26	26	24	20	22
Total tumor	10(52.6%)	21(80.8%)*	20(76.9%)	24(100%)*	18(90%)	21(95.5%)
TML	0(0%)	3(11.5%)	0(0%)	13(54.2%)***	1(5%)	13(59.1%)**
Adenoma in lung	8(42.1%)	9(34.6%)	17(65.4%)	7(29.2%)*	13(65%)	6(27.2%)*
AC in lung	2(10.5)	8(30.8%)	3(11.5%)	8(33.3%)	4(20%)	4(18.2%)
TML lung metastasis	0(0%)	3(11.5%)	0(0%)	7(29.2%)**	1(5%)	10(45.5%)**
AC in lung + TML lung metastasis	2(10.5%)	11(42.3%)	3(11.5%)	15(62.5%)***	5(25%)	14(63.7%)*
Total lung tumor	10(52.6%)	20(76.9%)	20(76.9%)	22(91.7%)	18(90%)	20(90.9%)
TML spleen metastasis	0(0%)	2(7.7%)	0(0%)	7(29.2%)**	1(5%)	6(27.2%)
TML liver metastasis	0(0%)	2(7.7%)	0(0%)	4(16.7%)*	1(5%)	7(31.8%)*
TML lymph node metastasis	0(0%)	2(7.7%)	0(0%)	1(4.2%)	1(5%)	7(31.8%)*
TML kidney Metastasis	0(0%)	2(7.7%)	0(0%)	6(25%)**	1(5%)	6(27.2%)*
TML heart metastasis	0(0%)	0(0%)	0(0%)	2(8.3%)	0(0%)	0(0%)
Subcutaneous fibrosarcoma	0(0%)	1(3.8%)	0(0%)	0(0%)	0(0%)	3(13.6%)

*P < 0.05;

**P < 0.01;

***P < 0.001, Significant difference between the FVB-Trp53^+/-^ and Wild-type mice in the same MNU administration group, as assessed using chi-square test. AC, adenocarcinoma in lung; TML, thymic malignant lymphoma.

**Fig 5:**
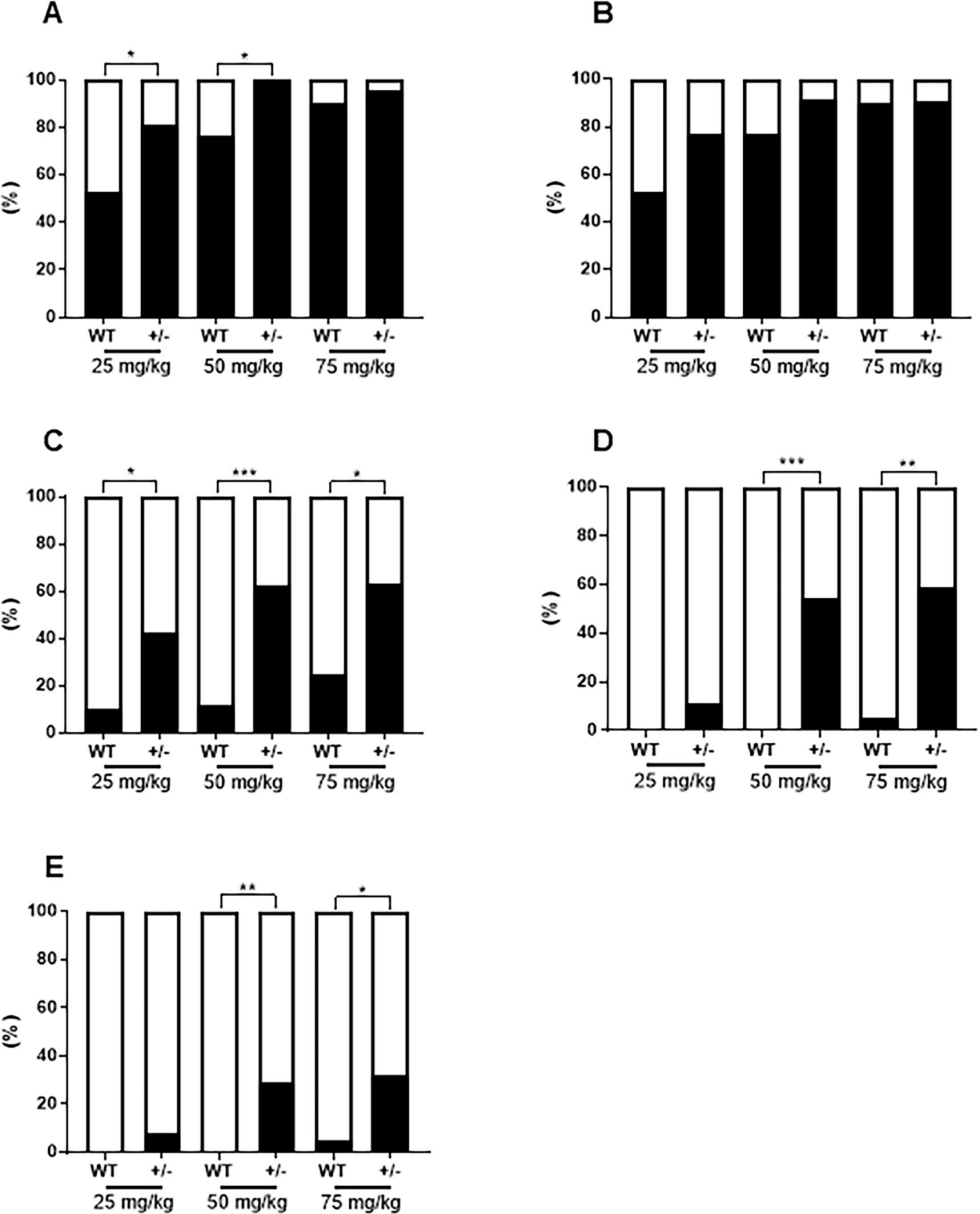
Tumor incidence rate (%) after MNU administration in different dosages. Tumor incidence in each group (A); the incidence of lung tumor (B); the incidence of adenocarcinoma and TML metastasis in the lungs (C); the incidence of thymic malignant lymphoma (TML) in the lungs; and the incidence of TML metastasis (E). *P < 0.05, **P < 0.01, ***P < 0.001 versus wild-type mice, as assessed using chi-square test.

The overall tumor incidence (Table 1) in FVB-Trp53^+/-^ mice was 80.8% and 100% in the 25 and 50 mg/kg groups, respectively, which was significantly higher than the 52.6% and 76.9% in wild-type mice (Fig 5A). Lung tumor incidence involving adenoma, adenocarcinoma, and TML lung metastasis in FVB-Trp53^+/-^ mice were 76.9%, 91.7%, and 90.9% in the 25, 50, and 75 mg/kg groups, respectively, which were higher than that of wild-type mice, but the difference was not significant (Fig 5B). The incidence rates of malignant tumors in the lungs of FVB-Trp53^+/-^ mice were 42.3%, 62.5%, and 63.7% in the 25, 50, and 75 mg/kg groups, respectively, which were significantly higher than the 10.5%, 11.5%, and 25.0% in wild-type mice (Fig 5C). The incidence rates of TML in the thymus of FVB-Trp53^+/-^ mice were 54.2% and 59.1% in the 50 mg/kg and 75 mg/kg groups, respectively, which were significantly higher than the 0% and 5% in wild-type mice (Fig 5D). Metastasis of TML was observed in 29.2% and 45.5% of FVB-Trp53^+/-^ mice in the 50 and 75 mg/kg groups, respectively, which was significantly higher than that in wild-type mice (0% and 5%, respectively) (Fig 5E).

### Immunohistochemistry

**Fig 6:**
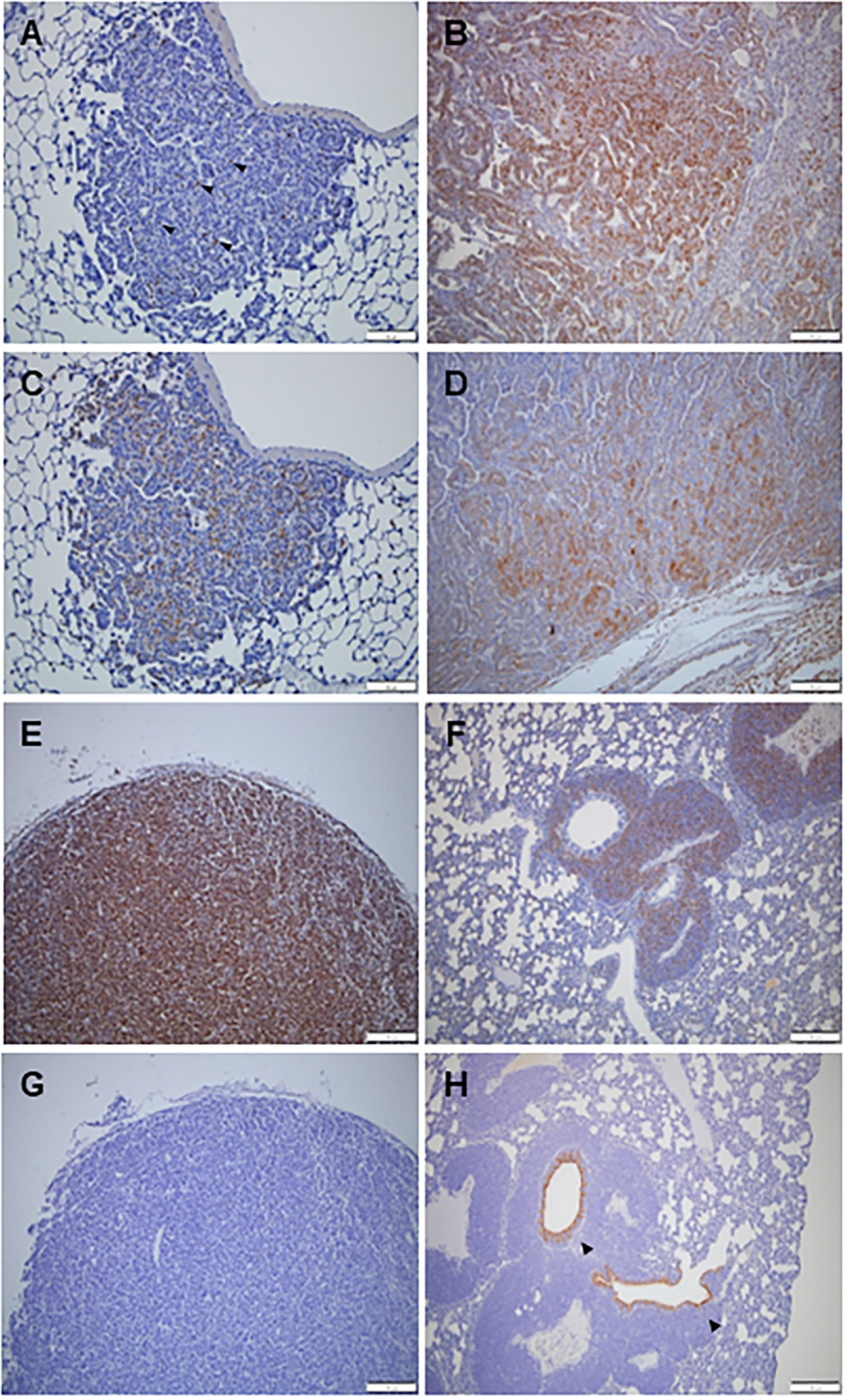
Representative images of immunohistochemical staining. A few PCNA-positive cells are shown in adenoma of the lung (A) Many PCNA-positive cells were found in lung adenocarcinoma (B), thymic malignant lymphoma (E), and thymic malignant lymphoma (TML) metastasis in the lungs (F). A few SPC-positive cells indicate an adenoma of the lungs (C), but many SPC-positive cells indicate adenocarcinoma of the lung (D). No CC10-positive cells were observed in the adenoma of the lungs (G), but CC10-positive cells were present in the bronchial epithelium at the center of the TML lung metastasis. Scale bar represents 100 *μ*m.

Immunohistochemical staining was performed to confirm the characteristics of the lung tumors. Proliferating cell nuclear antigen (PCNA), prosurfactant protein C (SPC), and CC10 (club cell 10-kDa protein) were used. PCNA is a cell proliferation marker that is stained in adenomas and adenocarcinomas of the lungs, TML, and TML lung metastases. In particular, malignant tumors, such as adenocarcinoma, TML, and TML lung metastases, were more strongly stained with PCNA (Fig 6B, 6E and 6F). In lung adenoma, PCNA was only expressed in some cells (Fig 6A). SPC, known as an alveolar type 2 cell marker, was observed in adenomas and adenocarcinomas in the lungs (Fig 6C and 6D). SPC staining was more strongly observed in adenocarcinomas than adenomas. However, SPC was not expressed in TML and TLM lung metastases. CC10, a Clara cell marker, was not expressed in adenoma and adenocarcinoma (Fig 6G) in the lungs and TML, but CC10 expression was observed in the bronchial epithelium at the center of TML lung metastasis (Fig 6H).

## Discussion

Transgenic mice used for short-term carcinogenicity tests are recognized for their value in drug validation and efficacy prediction through drug modeling of target genes [21, 22]. Using a mouse model for human disease, the safety and efficacy of the drug in vivo can be evaluated and predicted [2]. In particular, in the case of cancer, which has the highest mortality rate, the development of anticancer drugs is actively underway. Therefore, a safety evaluation using animal models is required. Tg-rasH2 [23] and B6-Trp53^+/-^ mice [24] are widely used in short-term carcinogenicity studies. Carcinogenesis involves mutations that occur in some genes of normal cells, thereby changing the properties of proteins that are the products of these genes. This leads to abnormal regulation of cell growth, which progresses to cancer [25]. p53 is a tumor suppressor gene that acts as a molecular link between the cause of cancer and the development of cancer [26]. This experiment was performed to determine whether FVB-Trp53^+/-^ mice, a model developed by TALAN-mediated gene targeting in Korea, is a suitable model for short-term carcinogenicity tests.

In a previous short-term carcinogenicity study, a single intraperitoneal injection of 75 mg/kg MNU as a positive carcinogen was used in B6-Trp53 heterozygous mice [27]. However, there is no data on short-term carcinogenicity tests using FVB-Trp53 mice. Therefore, in this study, to select the dose of MNU as a positive control in FVB-Trp53^+/-^ mice, 26-week short-term carcinogenicity was assessed after a single intraperitoneal injection of 25, 50, or 75 mg/kg. In previous studies of B6-Trp53^+/-^ male mice injected with 75 mg/kg MNU, only 6.7 [27] and 0% [17] of mice survived before the conclusion of the experiment. In the present study, 31.8% (7 of 22) of the FVB-Trp53^+/-^ male mice administered 75 mg/kg MNU survived until the end of the experiment.

When B6-Trp53^+/-^ male mice were injected with 75 mg/kg MNU, incidence of 26.7% thymic mass/nodules and 50% enlarged thymus in 50% was reported, and 80% of male mice had malignant lymphoma associated with abnormalities of the thymus [27]. A lower incidence (54.2 and 59.1%, respectively) of TML in both the 50 and 75 mg/kg groups was observed in FVB-Trp53^+/-^ male mice than in previous studies [17, 27]. Moreover, malignant lymphoma metastasized to various organs, such as the spleen, lymph nodes, liver, kidney, and lungs [17, 27], as observed in our study. In addition, TML metastasized to the lungs, spleen, lymph nodes, liver, kidney, and heart in FVB-Trp53^+/-^ male mice.

A higher incidence (> 40%) of malignant lung tumors was observed in the 25–75 mg/kg group in this study than in previous studies [17, 27]. Interestingly, lung adenocarcinoma was observed in 30.8, 33.3, and 18.2% of FVB-Trp53^+/-^ mice in the 25, 50, and 75 mg/kg groups, respectively. Furthermore, the incidence of primary lung tumors, such as adenoma and adenocarcinoma, which has not been reported in previous papers [17, 27], was 65.4, 62.5, and 45.4% in the 25, 50, and 75 mg/kg groups of FVB-Trp53^+/-^ mice, respectively, supporting the reports that FVB/N mice are susceptible to lung tumorigenesis [28, 29]. The main tumor types in FVB-Trp53^+/-^ mice were TML and primary lung tumors, regardless of the dose of MNU administered. However, the main tumor type in C57BL/6 background Trp53^+/-^ mice is malignant lymphoma in the thymus and spleen [17, 27, 30, 31].

Based on our data and a previously published paper, MNU could be used as a positive control for FVB-Trp53^+/-^ mice in short-term carcinogenicity studies. We found that both the 50 and 75 mg/kg groups of FVB-Trp53^+/-^ mice had similar incidences of TML and lung tumors. Furthermore, the survival rate in the 50 mg/kg group was higher than that in the 75 mg/kg group. Therefore, 50 mg/kg in FVB-Trp53^+/-^ mice was considered a suitable concentration for positive control in a short-term carcinogenic study.

Immunohistochemical staining was performed to investigate the pattern of primary lung tumors in FVB-Trp53 mice. PCNA is considered a key prognostic index for cancer [32]. PCNA in malignant tumors of the lungs was more prominently stained than in lung adenoma. CC10 is an anti-inflammatory protein produced by epithelial cells in the lungs, rarely found in human non-small cell carcinoma or tumor cell lines, and abundantly produced in progenitor cells of normal and neoplastic epithelium [33]. CC10 expression was observed only in the bronchiolar epithelium at the center of TML lung metastasis, while low CC10 expression was observed in primary adenoma or adenocarcinoma of the lungs. Nonetheless, it is difficult to conclude that anti-inflammatory response is increased in TML lung metastasis. SPC is a pulmonary surfactant protein C that is one of the four surfactant proteins produced by type II alveolar epithelial cells [34]. The expression of SPC decreases significantly in various types of lung injury and is associated with ACE apoptosis [35]. The primary adenoma and adenocarcinoma in the lungs in this study showed SPC expression, which indicates the possibility of a type II alveolar cell-derived primary lung tumor.

In summary, FVB-Trp53 heterozygous mice were used for short-term carcinogenicity tests, using MNU as a positive control. A single dose of 50 mg/kg MNU in FVB-Trp53^+/-^ might be a suitable concentration for positive control in short-term carcinogenic studies.

## Supporting information

S1 Table Hematology test results for 25, 50, 75 mg/kg administration group. (DOCX)

S2 Table Serum chemistry test results for 25, 50, 75 mg/kg administration group. (DOCX)

S1 Checklist Humane endpoints checklist. (DOCX)

## References

[1] Ito R, Takahashi T, Ito M. Humanized mouse models: Application to human diseases. Journal of Cellular Physiology. 2018;233(5):3723–8. doi: 10.1002/jcp.26045 28598567

[2] Jacobson-Kram D, Sistare FD, Jacobs AC. Use of transgenic mice in carcinogenicity hazard assessment. Toxicologic Pathology. 2004;32 Suppl 1:49–52. doi: 10.1080/01926230490424761 15209403

[3] Nambiar PR and Morton D. The rasH2 mouse model for assessing carcinogenic potential of pharmaceuticals. Toxicologic Pathology. 2013;41(8):1058–67. doi: 10.1177/0192623313477257 23423820

[4] Alexandrova EM, Mirza SA, Xu S, Schulz-Heddergott R, Marchenko ND, Moll UM. P53 loss-of-heterozygosity is a necessary prerequisite for mutant P53 stabilization and gain-of-function in vivo. Cell Death & Disease. 2017;8(3):e2661. doi: 10.1038/cddis.2017.80 28277540 PMC5386572

[5] Duffy MJ, Synnott NC, Crown J. Mutant p53 as a target for cancer treatment. European Journal of Cancer. 2017;83:258–65. doi: 10.1016/j.ejca.2017.06.023 28756138

[6] Kuperwasser C, Hurlbut GD, Kittrell FS, Dickinson ES, Laucirica R, Medina D, et al. Development of spontaneous mammary tumors in BALB/c p53 heterozygous mice: a model for Li-Fraumeni syndrome. American Journal of Pathology. 2000;157(6):2151–9. 11106587 10.1016/S0002-9440(10)64853-5 PMC1885755

[7] Levine AJ and Oren M. The first 30 years of p53: Growing ever more complex. Nature Reviews Cancer. 2009;9(10):749–58. doi: 10.1038/nrc2723 19776744 PMC2771725

[8] Yue X, Zhao Y, Xu Y, Zheng M, Feng Z, Hu W. Mutant p53 in cancer: Accumulation, gain-of-function, and therapy. Journal of Molecular Biology. 2017;429(11):1595–1606. doi: 10.1016/j.jmb.2017.03.030 28390900 PMC5663274

[9] Mello SS and Attardi LD. Deciphering p53 signaling in tumor suppression. Current Opinion in Cell Biology. 2018;51:65–72. doi: 10.1016/j.ceb.2017.11.005 29195118 PMC5949255

[10] Issaeva N. p53 signaling in cancers. Cancers. 2019;11(3):332. doi: 10.3390/cancers11030332 30857153 PMC6468470

[11] Yang D, Cheng D, Tu Q, Yang H, Sun B, Yan L, et al. HUWE1 controls the development of non-small cell lung cancer through down-regulation of p53. Theranostics. 2018;8(13):3517–29. doi: 10.7150/thno.24401 30026863 PMC6037029

[12] Chen J. The cell-cycle arrest and apoptotic functions of p53 in tumor initiation and progression. Cold Spring Harb Prospective in Medicine. 2016;6(3):a026104. doi: 10.1101/cshperspect.a026104 26931810 PMC4772082

[13] Petruska JM, Frank DW, Freeman GB, Evans EW, Macdonald JS. Toxicity and carcinogenicity studies of chlorpromazine hydrochloride and p-cresidine in the p53 heterozygous mouse model. Toxicologic pathology. 2002;30(6):696–704. doi: 10.1080/01926230290166788 12512871

[14] Long GG, Morton D, Peters T, Short B, Skydsgaard M. Alternative mouse models for carcinogenicity assessment: Industry use and issues with pathology interpretation. Toxicologic Pathology. 2010;38(1):43–50. doi: 10.1177/0192623309354107 19915137

[15] Faustino-Rocha AI, Ferreira R, Oliveira PA, Gama A, Ginja M. N-methyl-N-nitrosourea as a mammary carcinogenic agent. Tumour Biology. 2015;36(12):9095–117. doi: 10.1007/s13277-015-3973-2 26386719

[16] Huo X, Li Z, Zhang S, Li C, Guo M, Lu J, et al. Analysis of the expression level and methylation of tumor protein p53, phosphatase and tensin homolog and mutS homolog 2 in N-methyl-N-nitrosourea-induced thymic lymphoma in C57BL/6 mice. Oncology Letters. 2017;14(4):4339–48. doi: 10.3892/ol.2017.6721 28943948 PMC5592855

[17] Liu S, Lyu J, Li Q, Wu X, Yang Y, Huo G, et al. Generation of a uniform thymic malignant lymphoma model with C57BL/6J p53 gene deficient mice. Journal of Toxicologic Pathology. 2022;35(1):25–36. doi: 10.1293/tox.2021-0022 35221493 PMC8828615

[18] Wu X, Liu S, Lyu J, Zhou S, Yang Y, Wang C, et al. Endogenous controls of gene expression in N-methyl-N-nitrosourea-induced T-cell lymphoma in p53-deficient mice. BMC Cancer. 2017;17:545. doi: 10.1186/s12885-017-3536-6 28807016 PMC5557555

[19] Mitsumori K, Onodera H, Shimo T, Yasuhara K, Takagi H, Koujitani T, et al. Rapid induction of uterine tumors with p53 point mutations in heterozygous p53-deficient CBA mice given a single intraperitoneal administration of N-ethyl-N-itrosourea. Carcinogenesis. 2000;21(5):1039–42. 10783330 10.1093/carcin/21.5.1039

[20] Yun WB, Kim JE, Lee ML, Choi JY, Park JJ, Song BR, et al. Sensitivity to tumor development by TALEN-mediated Trp53 mutant genes in the susceptible FVB/N mice and the resistance C57BL/6 mice. Laboratory Animal Research. 2021;37:32. doi: 10.1186/s42826-021-00107-y 34839833 PMC8628475

[21] Tennant RW, French JE, Spalding JW. Identifying chemical carcinogens and assessing potential risk in short-term bioassays using transgenic mouse models. Environmental Health Perspectives. 1995;103(10):942–50. doi: 10.1289/ehp.95103942 8529591 PMC1519166

[22] Eastmond DA, Vulimiri SV, French JE, Sonawane B. The use of genetically modified mice in cancer risk assessment: Challenges and limitations. Critical Reviews in Toxicology. 2013; 43(8):611–31. doi: 10.3109/10408444.2013.822844 23985072 PMC4457504

[23] Suemizu H, Muguruma K, Maruyama C, Tomisawa M, Kimura M, Hioki K, et. Al. Transgene stability and features of rasH2 mice as an animal model for short-term carcinogenicity testing. Molecular Carcinogenesis. 2002;34(1):1–9. doi: 10.1002/mc.10045 12112317

[24] Donehowe LA, Harvey M, Slagle BL, McArthur MJ, Montgomery CA Jr, Butel JS, et al. Mice deficient for p53 are developmentally normal but susceptible to spontaneous tumours. Nature. 1992:356:215–21. doi: 10.1038/356215a0 1552940

[25] Parrales A and Iwakuma T. Targeting oncogenic mutant p53 for cancer therapy. Frontiers in Oncology. 2015;5:288. doi: 10.3389/fonc.2015.00288 26732534 PMC4685147

[26] Harris CC. P53 tumor suppressor gene: At the crossroads of molecular carcinogenesis, molecular epidemiology, and cancer risk assessment. Environmental Health Perspectives. 1996;104 Suppl 3(Suppl 3):435–9. doi: 10.1289/ehp.96104s3435 8781359 PMC1469608

[27] Morton D, Bailey KL, Stout CL, Weaver RJ, White KA, Lorenzen MJ, et al. N-methyl-N-nitrosourea (MNU): A positive control chemical for p53+/- mouse carcinogenicity studies. Toxicologic Pathology. 2008;36(7):926–31. doi: 10.1177/0192623308324959 18827072

[28] Mahler JF, Stokes W, Mann PC, Takaoka M, Maronpot RR. Spontaneous lesions in aging FVB/N mice. Toxicologic Pathology. 1996;24(6):710–716. doi: 10.1177/019262339602400606 8994298

[29] Malkinson AM. Inheritance of pulmonary adenoma susceptibility in mice. Progress in Experimental Tumor Research. 1999;35:78–94. doi: 10.1159/000062005 10377753

[30] Hoivik DJ, Allen JS, Wall HG, Nold JB, Miller RT, Santostefano MJ. Studies evaluating the utility of N-methyl-N-nitrosourea as a positive control in carcinogenicity studies in the p53+/- mouse. International Journal of Toxicology. 2005;24(5):349–56. doi: 10.1080/10915810500210385 16257854

[31] Reese JS, Allay E, Gerson SL. Overexpression of human O6-alkylguanine DNA alkyltransferase (AGT) prevents MNU induced lymphomas in heterozygous p53 deficient mice. Oncogene, 2001;20(38): 5258–63. doi: 10.1038/sj.onc.1204700 11536039

[32] Ye X, Ling B, Xu H, Li G, Zhao X, Xu J, et al. Clinical significance of high expression of proliferating cell nuclear antigen in non-small cell lung cancer. Medicine. 2020;99(16):e19755. doi: 10.1097/MD.0000000000019755 32311975 PMC7220128

[33] Linnoila RI, Szabo E, DeMayo F, Witschi H, Sabourin C, Malkinson A. The role of CC10 in pulmonary carcinogenesis: from a marker to tumor suppression. Annals of the New York Academy of Sciences. 2000;923:249–67. doi: 10.1111/j.1749-6632.2000.tb05534.x 11193761

[34] Darawshy F, Rmeileh AA, Kuint R, Berkman N. Possible association between SP-C mutations and lung cancer: Two case reports and review of literature. Cancer Treatment and Research Communications. 2021;29:100461. doi: 10.1016/j.ctarc.2021.100461 34600418

[35] Puthusseri B, Marudamuthu A, Tiwari N, Fu J, Idell S, Shetty S. Regulation of p53-mediated changes in the uPA-fibrinolytic system and in lung injury by loss of surfactant protein C expression in alveolar epithelial cells. Lung Cellular and Molecular Physiology. 2017;312:L783–L796. doi: 10.1152/ajplung.00291.2016 28385810 PMC5495940

