# Supplementary material for: Short-term carcinogenicity study of N-methyl-N-nitrosourea in FVB-Trp53 heterozygous mice
Source: PLoS One. 2023 Jan 6;18(1):e0280214. doi: 10.1371/journal.pone.0280214 (PMC9821506; doi:10.1371/journal.pone.0280214)
Supplement: S1 Table — (DOCX) [file pone.0280214.s001.docx]

**S1 Table 1. Hematology test results for 25, 50, 75 mg/kg administration group**

|  | 25mg/kg | | 50mg/kg | | 75mg/kg | |
| --- | --- | --- | --- | --- | --- | --- |
|  | p53^+/+^ | p53^+/-^ | p53^+/+^ | p53^+/-^ | p53^+/+^ | p53^+/-^ |
| Number of animals | 19 | 22 | 25 | 15 | 18 | 7 |
| WBC (10^3^/mm^3^) | 2.6 ± 1.6 | 3.3 ± 1.0 | 1.9 ± 0.8 | 2.4 ± 1.3 | 2.7 ± 1.1 | 2.0 ± 0.9 |
| RBC (10^6^/mm^3^) | 8.5 ± 1.1 | 8.8 ± 0.4 | 8.4 ± 0.5 | 9.0 ± 0.9^*^ | 9.0 ± 0.4 | 9.1 ± 0.4 |
| Hemoglobin (g/dl) | 11.4 ± 1.5 | 11.9 ± 0.8 | 11.5 ± 0.7 | 12.1 ± 1.4 | 12.2 ± 0.6 | 12.1 ± 0.4 |
| Hematocrit (%) | 38.7 ± 5.6 | 40.8 ± 2.3 | 39.2 ± 1.9 | 41.5 ± 4.8 | 40.7 ± 1.9 | 40.2 ± 1.2 |
| MCV (fl) | 45.7 ± 1.2 | 46.4 ± 1.0 | 46814.0 ± 0.9 | 45.4 ± 1.2 | 45.1 ± 0.9 | 44.2 ± 0.7^*^ |
| MCH (pg) | 13.5 ± 0.3 | 13.5 ± 0.4 | 13.8 ± 0.3 | 13.5 ± 0.3^*^ | 13.6 ± 0.3 | 13.3 ± 0.3 |
| MCHC (g/dl) | 29.5 ± 0.7 | 29.2 ± 0.8 | 29.5 ± 0.7 | 29.2 ± 0.6 | 30.0 ± 0.5 | 30.1 ± 0.3 |
| CHCM (g/dl) | 29.8 ± 0.7 | 29.3 ± 0.6 | 28.6 ± 0.6 | 28.8 ± 0.4 | 29.6 ± 0.5 | 30.0 ± 0.3 |
| RDW (%) | 14.5 ± 0.5 | 14.8 ± 0.8 | 14.5 ± 0.6 | 15.1 ± 2.6 | 13.7 ± 0.3 | 13.7 ± 0.5 |
| HDW (%) | 2.3 ± 0.1 | 2.3 ± 0.1 | 2.2 ± 0.1 | 2.3 ± 0.3 | 2.2 ± 0.1 | 2.2 ± 0.1 |
| CH (pg) | 13.6 ± 0.2 | 13.5 ± 0.3 | 13.3 ± 0.2 | 13.3 ± 0.3 | 13.3 ± 0.1 | 13.2 ± 0.2 |
| CHDW (pg) | 1.8 ± 0.1 | 1.8 ± 0.1 | 1.8 ± 0.1 | 1.8 ± 0.2 | 1.8 ± 0.0 | 1.8 ± 0.1 |
| Platelet (10^3^/mm^3^) | 1280.4 ± 344.8 | 1442.8 ± 368.2 | 1621.0 ± 263.8 | 1625.8 ± 257.0 | 1437.4 ± 325.9 | 1432.1 ± 161.3 |
| MPV (fl) | 6.5 ± 2.3 | 6.6 ± 1.4 | 7.8 ± 0.6 | 7.8 ± 0.5 | 5.6 ± 0.4 | 4.7 ± 0.2^*^ |
| PDW (%) | 73.6 ± 8.4 | 70.7 ± 13.5 | 77.6 ± 10.5 | 73.9 ± 5.1 | 61.4 ± 9.9 | 50.5 ± 4.3^*^ |
| Plateletcrit (%) | 0.8 ± 0.2 | 0.9 ± 0.2 | 1.3 ± 0.2 | 1.3 ± 0.2 | 0.8 ± 0.2 | 0.7 ± 0.1 |
| Neutrophil (10^3^/mm^3^) | 0.5 ± 0.5 | 0.9 ± 0.7 | 0.4 ± 0.2 | 0.7 ± 0.5^*^ | 0.6 ± 0.3 | 0.5 ± 0.2 |
| Neutrophil (%) | 15.2 ± 5.3 | 25.0 ± 14.7^*^ | 18.0 ± 5.9 | 29.9 ± 18.5^*^ | 25.7 ± 9.6 | 29.2 ± 11.1 |
| Lymphocyte (10^3^/mm^3^) | 2.0 ± 1.1 | 2.3 ± 0.9 | 1.5 ± 0.7 | 1.6 ± 1.0 | 1.8 ± 0.9 | 1.3 ± 0.8 |
| Lymphocyte (%) | 77.4 ± 6.5 | 69.0 ± 17.3 | 75.4 ± 7.0 | 64.5 ± 18.2^*^ | 67.4 ± 9.8 | 64.6 ± 10.0 |
| Monocyte (10^3^/mm^3^) | 0.1 ± 0.1 | 0.1 ± 0.1 | 0.1 ± 0.0 | 0.1 ± 0.0 | 0.1 ± 0.1 | 0.1 ± 0.1 |
| Monocyte (%) | 3.1 ± 1.3 | 2.8 ± 1.4 | 3.5 ± 1.6 | 2.7 ± 1.3 | 2.5 ± 1.0 | 2.6 ± 1.5 |
| Eosinophil (10^3^/mm^3^) | 0.1 ± 0.1 | 0.0 ± 0.0 | 0.0 ± 0.0 | 0.0 ± 0.0 | 0.1 ± 0.1 | 0.1 ± 0.0 |
| Eosinophil (%) | 2.7 ± 4.0 | 1.4 ± 0.8 | 1.7 ± 1.0 | 1.8 ± 1.8 | 3.6 ± 5.4 | 3.0 ± 1.4 |
| Basophil (10^3^/mm^3^) | 0.0 ± 0.0 | 0.0 ± 0.0 | 0.0 ± 0.0 | 0.0 ± 0.0 | 0.0 ± 0.0 | 0.0 ± 0.0 |
| Basophil (%) | 0.2 ± 0.2 | 0.2 ± 0.2 | 0.2 ± 0.1 | 0.2 ± 0.1 | 0.2 ± 0.1 | 0.1 ± 0.1 |
| LUC(10^3^/mm^3^) | 0.0 ± 0.1 | 0.1 ± 0.0 | 0.0 ± 0.0 | 0.0 ± 0.0 | 0.0 ± 0.0 | 0.0 ± 0.0 |
| LUC (%) | 1.4 ± 1.0 | 1.6 ± 0.9 | 1.2 ± 0.7 | 1.0 ± 0.6 | 0.6 ± 0.3 | 0.5 ± 0.2 |
| Reticulocyte (10^3^/mm^3^) | 395.6 ± 72.3 | 435.4 ± 84.6 | 50.13 ± 64.3 | 484.3 ± 205.2 | 343.9 ± 47.8 | 372.8 ± 53.3 |
| Reticulocyte (%) | 4.7 ± 0.6 | 5.0 ± 0.9 | 4.8 ± 0.8 | 5.5 ± 2.7 | 3.8 ± 0.4 | 4.1 ± 0.6 |

Mean ± S.D. (S.D., standard deviation) ^*^*P* < 0.05, significant difference between the FVB-Trp53^+/-^ and Wild-type mice type in the same MNU administration group.
