# Supplementary material for: Short-term carcinogenicity study of N-methyl-N-nitrosourea in FVB-Trp53 heterozygous mice
Source: PLoS One. 2023 Jan 6;18(1):e0280214. doi: 10.1371/journal.pone.0280214 (PMC9821506; doi:10.1371/journal.pone.0280214)
Supplement: S2 Table — (DOCX) [file pone.0280214.s002.docx]

**S2 Table 2.** **Serum chemistry test results for 25, 50, 75 mg/kg administration group**

|  | 25mg/kg | | 50mg/kg | | 75mg/kg | |
| --- | --- | --- | --- | --- | --- | --- |
|  | p53^+/+^ | p53^+/-^ | p53^+/+^ | p53^+/-^ | p53^+/+^ | p53^+/-^ |
| Number of animals | 19 | 22 | 25 | 15 | 18 | 7 |
| ALT(GPT, IU/L) | 72.7 ± 43.1 | 79.5 ± 39.1 | 96.1 ± 24.3 | 90.3 ± 48.5 | 48.4 ± 12.7 | 63.3 ± 55.8 |
| ALP(IU/L) | 191.6 ± 36.6 | 170.0 ± 45.2 | 202.5 ± 36.0 | 213.1 ± 51.4 | 239.8 ± 32.8 | 228.3 ± 34.8 |
| AST(GOT, IU/L) | 83.6 ± 46.1 | 119.8 ± 93.7 | 139.7 ± 91.8 | 178.0 ± 124.7 | 99.4 ± 25.3 | 155.3 ± 161.4 |
| γGT(IU/L) | 0.1 ± 0.3 | 0.1 ± 0.4 | 0.0 ± 0.0 | 0.0 ± 0.0 | 0.0 ± 0.0 | 0.0 ± 0.0 |
| Triglyceride(mg/dL) | 126.9 ± 33.4 | 89.6 ± 25.8^*^ | 90.6 ± 26.7 | 74.5 ± 21.8 | 142.2 ± 43.1 | 107.3 ± 76.2 |
| Albumin(g/dL) | 1.7 ± 0.1 | 1.7 ± 0.1 | 1.7 ± 0.1 | 1.7 ± 0.1 | 1.8 ± 0.1 | 17. ± 0.1 |
| Glucose(mg/L) | 283.8 ± 44.7 | 263.5 ± 45.4 | 297.3 ± 60.5 | 271.8 ± 56.7 | 109.3 ± 26.0 | 145.1 ± 40.1^*^ |
| A/G ratio | 0.5 ± 0.0 | 0.5 ± 0.0 | 0.5 ± 0.0 | 0.5 ± 0.0 | 0.6 ± 0.1 | 0.6 ± 0.1 |
| Total protein(g/dL) | 4.9 ± 0.3 | 4.8 ± 0.3 | 5.0 ± 0.3 | 5.1 ± 0.4 | 4.9 ± 0.2 | 4.7 ± 0.4 |
| TC(mg/dL) | 165.6 ± 28.6 | 17.0 ± 39.1 | 181.3 ± 25.8 | 180.8 ± 36.5 | 168.5 ± 32.1 | 155.0 ± 14.6 |
| Total bilirubin(mg/dL) | 0.1 ± 0.0 | 0.1 ± 0.0 | 0.1 ± 0.0 | 0.1 ± 0.0 | 0.1 ± 0.0 | 0.1 ± 0.0 |
| BUN(mg/dL) | 17.0 ± 4.3 | 17.8 ± 5.1 | 19.3 ± 4.0 | 18.2 ± 4.8 | 23.2 ± 4.7 | 29.2 ± 20.0 |
| Creatinine(mg/dL) | 0.3 ± 0.1 | 0.3 ± 0.1 | 0.3 ± 0.1 | 0.3 ± 0.1 | 0.3 ± 0.0 | 0.3 ± 0.0 |
| B/C ratio | 55.9 ± 13.8 | 58.8 ± 10.8 | 61.7 ± 11.7 | 55.0 ± 15.6 | 89.4 ± 17.3 | 108.5 ± 70.3 |
| Calcium(mg/dL) | 9.2 ± 0.3 | 9.3 ± 0.4 | 9.3 ± 0.4 | 9.5 ± 0.5 | 8.9 ± 0.2 | 8.7 ± 0.2^*^ |
| Chlorine(mEq/L) | 111.0 ± 2.1 | 110.9 ± 2.3 | 110.2 ± 2.2 | 111.4 ± 2.7 | 116.8 ± 2.4 | 115.4 ± 1.5 |
| IP(mg/dL) | 7.7 ± 0.1 | 8.8 ± 1.2^*^ | 9.1 ± 2.0 | 9.3 ± 1.1 | 7.2 ± 0.7 | 8.3 ± 1.3^*^ |
| Potassium(mEq/L) | 5.3 ± 0.6 | 5.5 ± 0.6 | 5.3 ± 1.0 | 5.5 ± 0.7 | 5.6 ± 0.7 | 5.5 ± 0.6 |
| Sodium(mEq/L) | 149.8 ± 1.2 | 150.3 ± 1.6 | 150.5 ± 2.3 | 152.6 ± 4.3 | 154.9 ± 1.5 | 154.3 ± 2.6 |

Mean ± S.D. (S.D., standard deviation) ^*^*P* < 0.05, significant difference between the FVB-Trp53^+/-^ and Wild-type mice in the same MNU administration group.
